# Re-analysis of an outbreak of Shiga toxin-producing *Escherichia coli* O157:H7 associated with raw drinking milk using Nanopore sequencing

**DOI:** 10.1038/s41598-024-54662-0

**Published:** 2024-03-09

**Authors:** David R. Greig, Vivienne Do Nascimento, David L. Gally, Saheer E. Gharbia, Timothy J. Dallman, Claire Jenkins

**Affiliations:** 1 National Infection Service, United Kingdom Health Security Agency, London, NW9 5EQ UK; 2 NIRH Health Protection Research Unit for Gastrointestinal Pathogens, Liverpool, UK; 3 Division of Infection and Immunity, The Roslin Institute and Royal (Dick) School of Veterinary Studies, University of Edinburgh, Easter Bush, Edinburgh, UK; 4 NIHR Health Protection Research Unit in Genomes and Enabling Data, Warwick, UK; 5 Institute for Risk Assessment Sciences (IRAS), Faculty of Veterinary Medicine, Utrecht University, 3584 CL Utrecht, The Netherlands

**Keywords:** Foodborne outbreak, Genomic epidemiology, STEC O157:H7, WGS, Nanopore, Illumina, Prophage comparison, Bacterial genetics, Genome informatics

## Abstract

The aim of this study was to compare Illumina and Oxford Nanopore Technology (ONT) sequencing data to quantify genetic variation to assess within-outbreak strain relatedness and characterise microevolutionary events in the accessory genomes of a cluster of 23 genetically and epidemiologically linked isolates related to an outbreak of Shiga toxin-producing *Escherichia coli* O157:H7 caused by the consumption of raw drinking milk. There were seven discrepant variants called between the two technologies, five were false-negative or false-positive variants in the Illumina data and two were false-negative calls in ONT data. After masking horizontally acquired sequences such as prophages, analysis of both short and long-read sequences revealed the 20 isolates linked to the outbreak in 2017 had a maximum SNP distance of one SNP between each other, and a maximum of five SNPs when including three additional strains identified in 2019. Analysis of the ONT data revealed a 47 kbp deletion event in a terminal compound prophage within one sample relative to the remaining samples, and a 0.65 Mbp large chromosomal rearrangement (inversion), within one sample relative to the remaining samples. Furthermore, we detected two bacteriophages encoding the highly pathogenic Shiga toxin (Stx) subtype, *Stx2a*. One was typical of *Stx2a*-phage in this sub-lineage (Ic), the other was atypical and inserted into a site usually occupied by *Stx2c*-encoding phage. Finally, we observed an increase in the size of the pO157 IncFIB plasmid (1.6 kbp) in isolates from 2019 compared to those from 2017, due to the duplication of insertion elements within the plasmids from the more recently isolated strains. The ability to characterize the accessory genome in this way is the first step to understanding the significance of these microevolutionary events and their impact on the genome plasticity and virulence between strains of this zoonotic, foodborne pathogen.

## Introduction

Shiga toxin-producing *Escherichia coli* (STEC) O157:H7 is a zoonotic, gastrointestinal pathogen that colonises the gut of healthy ruminants, particularly cattle and sheep^1,2^. Transmission to humans occurs through consumption of contaminated food or water, direct or indirect contact with animals or their environment. STEC O157:H7 infection in humans can produce a wide range of symptoms, from mild diarrhoea to severe bloody diarrhoea, abdominal cramps and vomiting^3^. In 5–15% of cases, the infection can lead to the development of haemolytic uremic syndrome (HUS), a severe multi-system syndrome, that can be fatal, particularly in young children and the elderly^4,5^.

STEC O157:H7 characteristically harbours one or more bacteriophage encoded Shiga toxin genes (*stx*), belonging to one of three toxin subtypes, *stx1a, stx2a* and/or *stx2c*. Subtype *stx2a* is associated with causing HUS^6,7^. STEC O157:H7 is divided into three lineages (I, II and I/II) and seven sub-lineages (Ia-Ic, IIa-IIc and I/II). Previously studies have shown that STEC O157:H7 belonging to sub-lineage Ic harbouring *stx2a* with or without *stx2c* (for the most part corresponding with phage type (PT) 21/28) was the dominant type in the UK between 1995 and 2015 and remains a common cause of STEC-HUS in England^3,6,8,9^.

Public health surveillance of STEC O157:H7 in England, including outbreak detection and investigation, is co-ordinated by UK Health Security Agency (UKHSA), formerly Public Health England (PHE). The National Enhanced STEC Surveillance System (NESSS) integrates short-read whole genome sequencing of STEC isolates from patients with symptoms of gastrointestinal disease, with epidemiological data capturing their food and travel histories, contact with animals and other environmental exposures. The use of short-read WGS data during outbreak investigations delivers an unprecedented level of strain discrimination, facilitates case ascertainment even when epidemiological links are obscured, and provides insight into the evolutionary context for emerging pathogenic strains^9–11^. Genetic relatedness of the sequences of the isolates is determined by reference-based variant calling to determine high-quality single nucleotide polymorphisms (SNPs)^12^. Previous studies have shown that isolates with genome sequences that fall within the same 5 SNP single linkage cluster (where all samples in the cluster are linked by 5 SNPs or less) are likely to be associated with the same source^9,11,13^.

The genome of STEC O157:H7 is approximately 5.5 Mbp in size^14^ and has a relatively small core genome size across the population due to a large and diverse accessory genome including the presence of large lambdoid prophages which makes up 10–15% of the STEC chromosome^15,16^. Due to the limitations of short read sequencing technologies in handling the homologous prophage content of the STEC O157:H7 chromosome, information and context regarding inter and intra variation in prophages, structural variation and even context surrounding plasmid content is lost. With the recent development of single-molecule real time sequencing (SMRT) technologies we now have the tools to de novo assemble pathogens into individual contigs containing a single replicon^17^ enabling us to characterise and scrutinise the accessory genome, including prophage sequences of STEC^17–21^.

We re-analysed the human, food and animal isolates linked to an outbreak of STEC serotype O157:H7 that occurred in 2017 on the Isle of Wight, caused by the consumption of raw drinking milk (RDM)^22^, using Nanopore sequencing data. In 2019, two additional cases infected with STEC O157:H7 that fell within the same 5-SNP single linkage cluster were detected^11^. These cases were resident in the same geographical location as the farm implicated in the outbreak investigation in 2017. The aim of the study was to evaluate our methodological approach to the analysis of isolates sequenced using the Oxford Nanopore Technology (ONT) platform and to assess the accessory genome variation between isolates within an outbreak attributed to a point source exposure, and between the isolates from the cases in 2019 that were geographically related but temporally distinct.

## Results and discussion

### Comparison of variant calling methods

**Figure 1:**
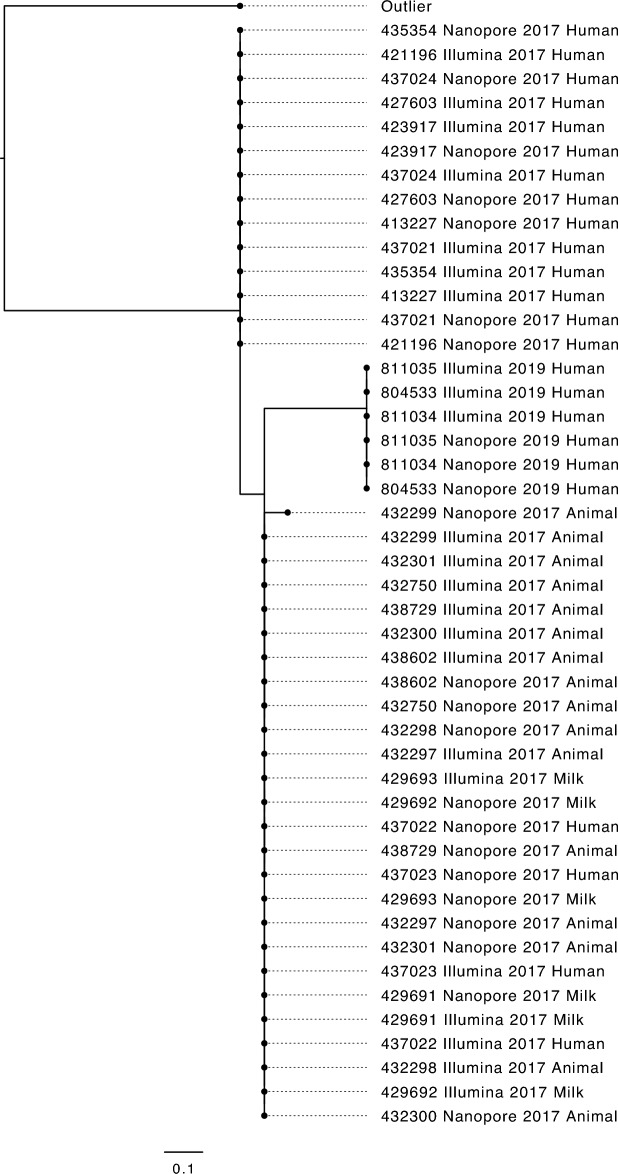
A maximum-likelihood phylogeny showing both Illumina derived and nanopore derived SNP-typing results for samples sequenced in this study.

Variant calling of the Illumina sequencing data identified 23 isolates that fell within the same 5-SNP single linkage cluster. The 20 isolates linked to the outbreak in 2017 had a maximum SNP distance of 1 SNP between each other, and a maximum of 5 SNPs when including cases identified in 2019 (Fig. 1). Analysis of the Nanopore data identified a maximum of 1 SNP variants between the 20 outbreak isolates and 5 SNPs between all 23 isolates (Fig. 1).

The variants identified in the Illumina sequencing data were compared to those identified in the Nanopore data. There were seven discrepant variants called between the two technologies (Table 1). Four of the seven mismatches were determined to be false positive variants in the Illumina dataset due to ambiguously aligning short reads (Table 1). A fifth, this time false negative, variant was called in the Illumina short read data in a single sample due to low coverage over a region of the genome where the variant was called (Table 1). The remaining two discrepant variants were deemed to be false negatives in the Nanopore data due to errors associated with calling SNPs within homopolymer sequences (Table 1).

**Table 1:** The discrepant variant calls between illumina and nanopore datasets for all outbreak samples.

Reference position	Reference base	Variant called	CDS	Locus Tag	Encodes	Count of samples with this discrepant variant (out of 23)	Reason for discrepancy (False positive/False negative)
232,300	G	A	NON CODING		n/a	1	Low coverage at position from Illumina data (FN)
270,579	A	G	R1168G	ECs0237	RhsG core protein with extension	2	Ambiguous alignment at this position from Illumina data (FP)
270,595	C	A	T1173N	ECs0237	RhsG core protein with extension	20	Ambiguous alignment at this position from Illumina data (FP)
379,516	A	G	NON CODING		n/a	4	Variant missed in homopolymer sequence by Nanopore data (FN)
418,442	T	G	E65D	ECs0395	thiogalactoside acetyltransferase	5	Variant missed in homopolymer sequence by Nanopore data (FN)
1,681,338	C	G	R314G	ECs1685	alanine racemase 2	20	Ambiguous alignment at this position from Illumina data (FP)
1,681,339	G	C	R314P	ECs1685	alanine racemase 2	20	Ambiguous alignment at this position from Illumina data (FP)

When accounting for the above false positive/false negative discrepant variants there remained only a single discrepant variant which was classified as a true variant [Nanopore (G var/T reference), Illumina (N/T reference)], accounting for the single SNP difference in sample 432,299 (Fig. 1).

This comparison highlighted the systemic differences associated with each technology, specifically the base-calling errors related to homopolymer detection observed in Nanopore data^23,24^ and the ambiguous alignment of Illumina data to homologous and paralogous regions^25^. This comparison also demonstrates the importance of masking these regions within the reference genome to produce accurate and meaningful results^23^.

### Genomic features of long-read assemblies of the chromosome

The assemblies of the sequences of the 23 isolates in this study all contained either two or three closed contigs, each supporting a single replicon (Table 2). The chromosome size of the isolates sequenced in this study (n = 23) ranged from 5,507,151 to 5,555,878 bp in length, a maximum difference of 48,727 bp and an average size of 5,553,177 bp (Table 2).

**Table 2:** Table detailing the ID of each strain, finalised assembly metrics, plasmid replicon typing, prophage counts and assembly-based accessions.

Strain ID	Source	# of contigs	Chromosome size (bp)	Total genome size (bp)	Plasmid 1 size (bp) and Inc group	Plasmid 2 size (bp) and Inc group	# of prophages	GenBank accession for chromosome	GenBank accessions for plasmids
413,227	Human	2	5,555,475	5,649,172	93,697 IncFIB	–	17	CP088060	CP088061
421,196	Human	2	5,555,066	5,649,123	94,057 IncFIB	–	17	CP088058	CP088059
423,917	Human	2	5,555,034	5,648,723	93,689 IncFIB	–	17	CP088056	CP088057
427,603	Human^a^	2	5,555,537	5,649,596	94,059 IncFIB	–	17	CP088071	CP088072
429,691	Milk	2	5,555,094	5,649,152	94,058 IncFIB	–	17	CP088054	CP088055
429,692	Milk	2	5,558,855	5,652,943	94,088 IncFIB	–	17	CP088052	CP088053
429,693	Milk	2	5,555,560	5,649,616	94,056 IncFIB	–	17	CP088050	CP088051
432,297	Animal	2	5,554,755	5,648,807	94,052 IncFIB	–	17	CP088048	CP088049
432,298	Animal	2	5,554,878	5,648,605	93,727 IncFIB	–	17	CP088046	CP088047
432,299	Animal	2	5,507,151	5,601,209	94,058 IncFIB	–	17	CP088044	CP088045
432,300	Animal	2	5,555,280	5,649,335	94,055 IncFIB	–	17	CP088042	CP088043
432,301	Animal	2	5,554,492	5,648,544	94,052 IncFIB	–	17	CP088040	CP088041
438,729	Animal	2	5,555,235	5,649,147	93,912 IncFIB	–	17	CP088069	CP088070
432,750	Animal	2	5,555,568	5,649,625	94,057 IncFIB	–	17	CP088067	CP088068
438,602	Animal	2	5,555,032	5,649,087	94,055 IncFIB	–	17	CP088038	CP088039
435,354	Human	3	5,555,067	5,735,080	94,051 IncFIB	85,962 (IncI1-γ)	17	CP088064	CP088065 + CP088066
437,021	Human^a^	2	5,555,314	5,649,370	94,056 IncFIB	–	17	CP088062	CP088063
437,022	Human ^b^	2	5,554,737	5,648,789	94,052 IncFIB	–	17	CP088036	CP088037
437,023	Human ^b^	2	5,554,791	5,648,842	94,051 IncFIB	–	17	CP088034	CP088035
437,024	Human^a^	2	5,555,270	5,649,330	94,060 IncFIB	–	17	CP088032	CP088033
804,533	Human	2	5,554,866	5,650,235	95,369 IncFIB	–	17	CP088030	CP088031
811,034	Human ^c^	2	5,555,205	5,650,573	95,368 IncFIB	–	17	CP088028	CP088029
811,035	Human ^c^	2	5,554,812	5,650,179	95,367 IncFIB	–	17	CP088026	CP088027

^a^, ^b^ and ^c^detail strains shared by the same case/patient.

**Figure 2:**
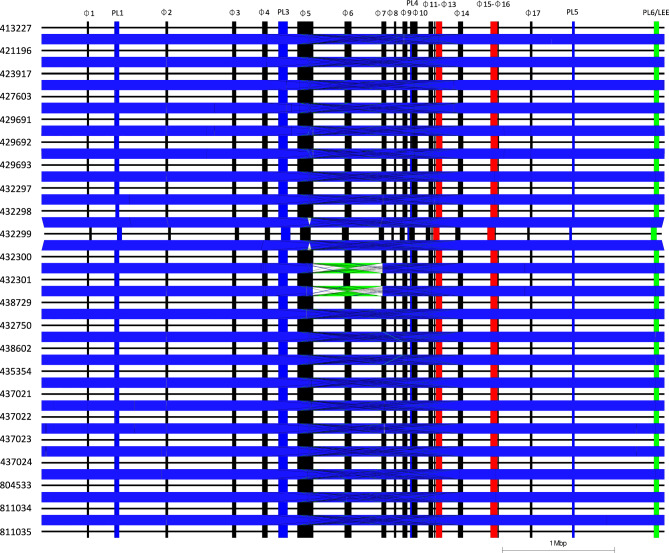
Easyfig alignment showing the chromosome and loci of prophages in all samples sequenced in this study. *Stx*-encoding prophage, Red; Prophage-like region, Blue; Locus of Enterocyte Effacement (LEE), Green and other non-*stx*-encoding prophages, Black.

Aligning and comparing the chromosomes of all 23 isolates within the same 5-SNP single linkage cluster led to the discovery of a large-scale recombination (LCR) event present in one of the cattle isolates (432,301) (Fig. 2). The large-scale recombination event was characterised by a 650kbp inversion between prophages 5 (*potC*) and 7 (*yebW*) (Fig. 2). At either edge of the inversion, prophages 5 (*potC*) and 7 (*yebW*) both have a 10.05kbp homologous sequence containing prophage structure encoding genes such as tail proteins, host specificity proteins and several hypothetical genes. The 10.05kbp homologous sequences in both prophages share a 97.7% sequence similarity.

It is known that the STEC O157:H7 genome undergoes large scale recombination to produce large inverted sequences within the chromosome^26,27^. The outbreak in this study was selected because it included eight isolates from cattle, and we wanted to look for LCRs in STEC O157:H7 in the animal reservoir, as well as in the RDM and human cases. We observed a LCR in just one cattle isolate; it is uncertain whether this genetic event occurred in vivo or on sub-culture in the laboratory. Potential phenotypic effects of LCRs, for example strain fitness, infectivity, or impact on patient outcomes, are yet to be determined. Within this 5-SNP cluster of 23 isolates, minimal large-scale chromosomal variation was observed, regardless of the source (animal, food or human), the clinical outcome of the case or the year the cases were detected.

### Analysis of prophage and prophage-like content

**Figure 3:**
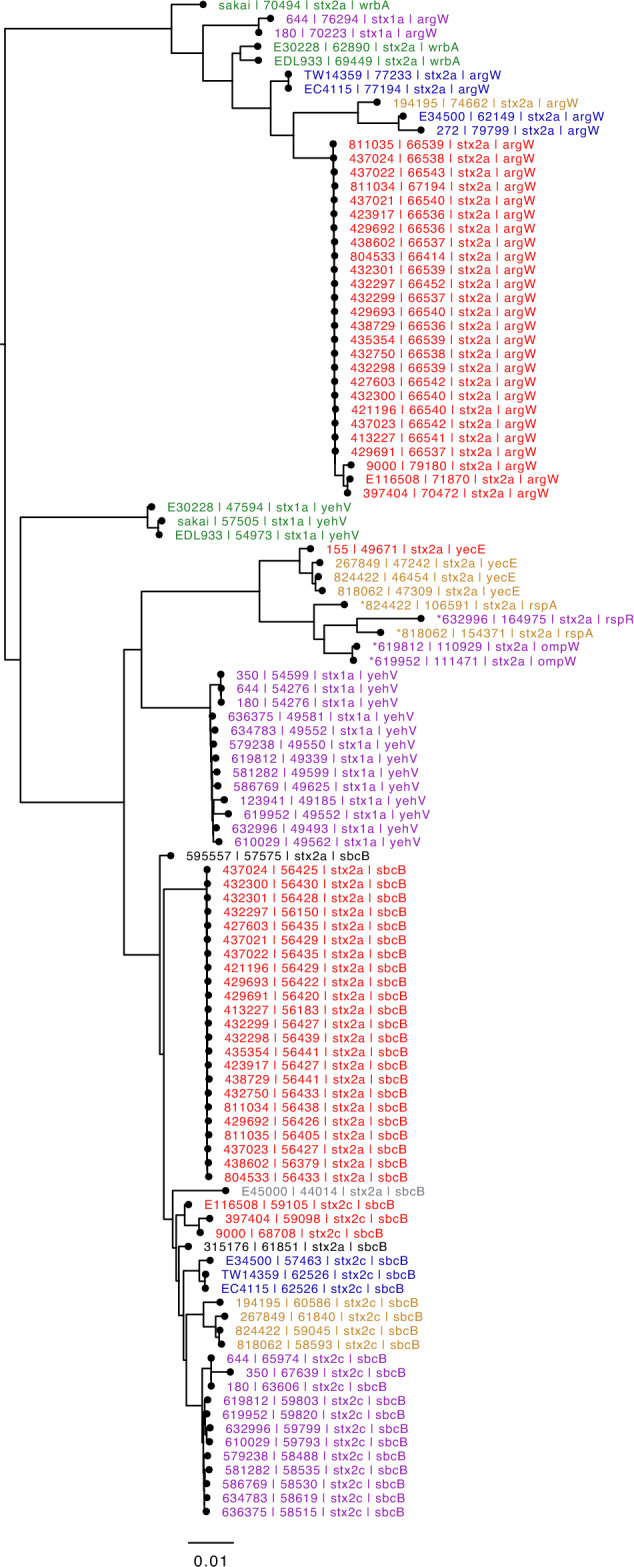
Neighbour joining tree based on Jaccard distances of *stx*-encoding prophages of publicly available samples and the outbreak samples sequenced in this study. Prophages are coloured by sub-lineage of STEC O157:H7. Sub-lineage Ia, Green; Ib, Yellow; Ic, Red; I/IIa, Blue; I/IIb, Grey; IIa, Orange; IIb, Black and IIc, Purple.

**Figure 4:**
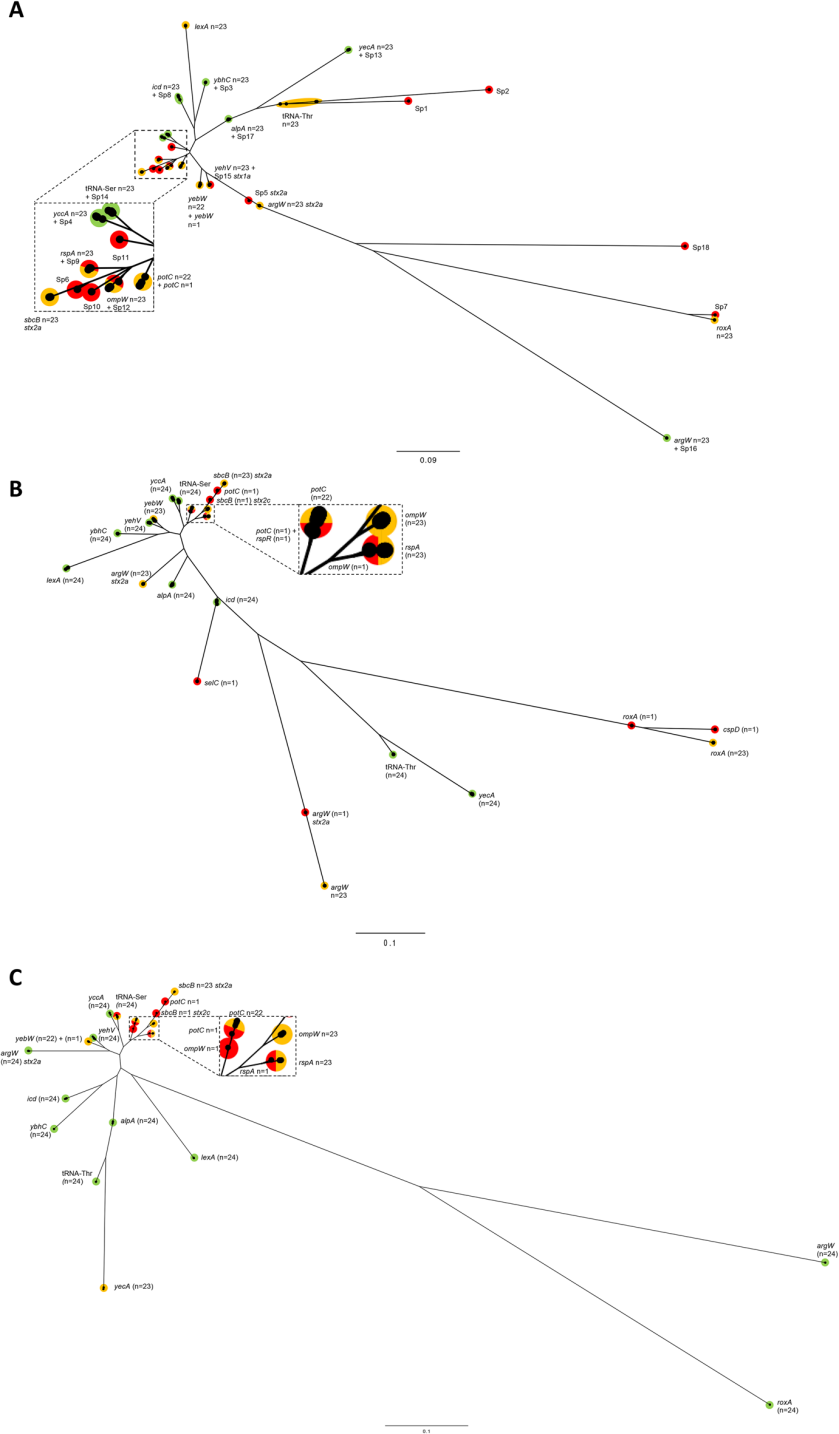
Mid-rooted neighbour-joining trees of Jaccard distances showing prophages from samples sequenced in this study with prophages from BA000007 (Sakai) (**A**), STEC O157:H7 strain 9000 (**B**) and STEC O157:H7 strain 397,404 (**C**). In each diagram prophages grouped by green are prophages shared in samples and reference genome; red are reference genome only and yellow are prophages unique samples sequenced in this study.

All 23 isolates contained the same number of prophages (n = 17) of which two were *stx2a*-encoding prophage (Figs. 2, 3). All prophages in the samples sequenced ranged from 8.2 to 144.5 kbp in size (Table S1). Across all 23 samples, 15/17 prophages were considered the same in all samples including prophages 1 (*lexA*), 2 (tRNA-Thr), 3 (*ybhC*), 4 (*yccA*), 6 (*rspA*). 8(*yecA*), 9 (tRNA-Ser), 10 (*ompW*), 11 (*icd*), 12 (*roxA*), 13 (*sbcB* [*stx2a*]), 14 (*yehV*), 15 (*argW* [*stx2a*]), 16 (*argW*) and 17 (*alpA*) (Figs. 2,4).

**Figure 5:**
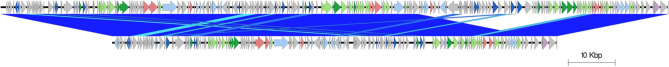
Easyfig alignment of compound prophage 5 with sample 432,300 (top) acting as a reference. Sample 432,299 (bottom) detailing a 47.3kbp deletion.

There were 2/17 prophages that showed variation, the first type of variation was a single deletion of a 47,389bp region in a large compound prophage 5 (*potC*) in a cattle isolate (432,299), different to the isolate exhibiting the LCR (Figs. 2, 4, 5). The deletion event was surrounded by two 5.8 kbp homologous sequences containing structural tail proteins and hypothetical genes. The second type of variation observed in prophages 5 (*potC*) and 7 (*yebW*) were related to the 0.65 Mbp LCR observed where these prophages acted as the break points.

Comparing the prophages identified in the outbreak strains to those identified in a well characterised STEC O157:H7 reference genome, strain Sakai (BA000007) isolated in Japan 30 years ago, it was noted that seven prophages matched between all outbreak genomes and the reference genome and they all shared the same integration sites. Those prophages included prophage 3 and Sp3 (*ybhC*), prophage 4 and Sp4 (*yccA*), prophage 11 and Sp8 (*icd*), prophage 8 and Sp13 (*yecA*), prophage 9 and Sp14 (tRNA-Ser), prophage 16 and Sp16 (*argW*), prophage 17 and Sp17 (*alpA*/*ssrA*) (Figs. 2, 4A).

There were three sets of prophages that had similar regions, including prophage 6 (*rspA*) to Sp9, prophage 10 (*ompW*) to Sp12 and prophage 14 (*yehV*) to Sp15 (*stx1a*) (Figs. 2, 4A). There were a further five prophages that were unique to all 23 samples described in this study, including prophage 1 (*lexA*), prophage 2 (tRNA-Thr), prophage 12 (*roxA*), prophage 13 (*sbcB* [*stx2a*]) and prophage 15 (*argW* [*stx2a*]) (Figs. 2, 4). A further two prophages showed variation within the 23 samples sequenced in this study but did not match any prophages found in strain Sakai, including prophages 5 (*potC*) and 7 (*yebW*) (Figs. 2, 4A). Finally, Sakai prophages Sp1, Sp2, Sp5 (*stx2a*), Sp6, Sp7, Sp10, Sp11 and Sp18 were all unique to the reference strain (Fig. 4A).

Comparing the prophages identified in the outbreak strains to those identified in a another well characterised STEC O157:H7 UK reference genome, strain 9000 (CP018252 - 2002) which is from the same sub-lineage (Ic) and phage type (PT21/28). It was noted that nine prophages were a match between the outbreak genomes and strain 9000. Those prophages included prophage 1 (*lexA*), prophage 2 (tRNA-Thr), prophage 3 (*ybhC*), prophage 4 (*yccA*), prophage 8 (*yecA*), prophage 9 (tRNA-Ser), prophage 11 (*icd*), prophage 14 (*yehV*) and prophage 17 (*alpA*/*ssrA*) (Fig. 4B).

Finally, comparing the prophages identified in the outbreak strains compared to those identified in a recent temporally concordant outbreak in 2017 linked to handling raw pet food in the UK^28^, from the same sub-lineage and phage type, strain 397,404 (CP043019). It was noted that ten prophages matched between all outbreak genomes and those from 397,404. Those prophages included prophage 1 (*lexA*), prophage 2 (tRNA-Thr), prophage 3 (*ybhC*), prophage 4 (*yccA*), prophage 11 (*icd*), prophage 12 (*roxA*), prophage 14 (*yehV*), prophage 15 (*argW* + *stx2a*), prophage 16 (*argW*) and prophage 17 (*alpA*/*ssrA*) (Fig. 4C).

Here, and in previous studies, pairwise comparisons revealed several common non-*stx*-encoding prophages found in strains of in STEC O157:H7 that are temporally and geographically distinct^21^. This indicates that certain prophages are stable within the STEC O157:H7 genome and perhaps can no longer be induced. However, in contrast to the above, some prophages which have previously been described as non-inducible have been shown to be mobile^14,16^. In the limited dataset included in our analysis to date, strains from the same sub-lineage and those more closely in time and space, had more prophages in common than those strains that were phylogenetically, geographically and temporally distinct^21,28^. Loss and acquisition of prophage content may be influenced by both time and geographical setting.

### Analysis of Shiga toxin-encoding prophages

All 23 isolates in the outbreak cluster harboured two *stx2a*-encoding prophages (Figs. 2, 3) previously undetected via Illumina sequencing; one of which was integrated at the *stx*-encoding bacteriophage integration site (SBI) *argW*, while the other was integrated at *sbcB*. The *stx2a*-encoding bacteriophage in sub-lineage Ic is commonly found at *argW*, and phylogenetic analysis showed that this bacteriophage clustered with *stx2a*-encoding phage within sub-lineage Ic (Fig. 3)^21^. The *sbcB* SBI is more commonly occupied by *stx2c*-encoding bacteriophages, and phylogenetic analysis showed that the *stx2a*-encoding bacteriophage integrated at *sbcB* in the outbreak strain was located on a branch mainly comprising *stx2c*-encoding bacteriophage^21^.

Previous studies have described the loss of *stx2c*-encoding phage and subsequent acquisition of *stx2a*-encoding phage exhibiting similar sequences to *stx2c*-encoding bacteriophage at the same SBI, in sub-lineage IIb^10,21^, however, this is the first report of this phenomenon occurring in sub-lineage Ic. Strains of STEC O157:H7 harbouring more than one *stx2a* prophage have been described previously^29^, but again this is the first report of the acquisition of two different *stx2a*-encoding phage in this sub-lineage. We previously showed that strains harbouring *stx2a* only belonging to sub-lineage Ic are significantly more likely to be associated with severe clinical outcomes than those strains harbouring *stx2a* only in sub-linage IIb^6^. The representative strains of STEC O157:H7 sub-lineage IIb in previous studies^10^ had only one type of *stx2a*-encoding phage; the presence of two different *stx2a*-encoding phage may play a role in enhancing pathogenicity in sub-lineage Ic.

### Plasmid analysis

**Figure 6:**
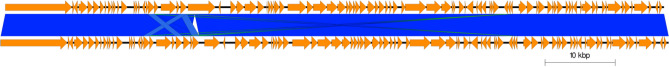
Easyfig alignment showing exemplar IncFIB plasmids from samples 432,301 (top) and 811,035 (bottom).

All isolates contained an IncFIB plasmid, the pO157 that is characteristic of STEC O157:H7, ranging in size from 93,689 to 94,050 between the outbreak isolates from 2017, a maximum difference of 361bp and an average size of 93,997bp. In the three temporally distinct isolates from 2019, the size range increased to 95,369bp with a maximum difference of 1,680bp. The approximately 1.6kbp increase between IncFIB plasmids in isolates from 2019 compared to those from 2017, is due to the duplication of insertion elements within those plasmids (Fig. 6). One isolate (435,354) from 2017, also contained a smaller IncI1-γ plasmid, 85,962by in size (Table 2a).

## Conclusions

In this study, we evaluated our bioinformatics approach to analysing long read sequencing data in an outbreak setting and showed the results of these analyses correlated well with the bioinformatics pipelines routinely employed for analysing the short-read sequencing data. Minimal LCRs and/or prophage variation was observed within the isolates linked to this point source outbreak of STEC O157:H7 PT21/28 caused by the consumption of RDM. Whether this is typical of small, geographically restricted, point source outbreaks of STEC, or characteristic of the microbiology of this specific strain and/or the epidemiological context of this setting remains to be seen. Given the association of *stx2a* with the potential to cause HUS, the discovery that the loss of *stx2c* in this strain has been followed by the acquisition of an additional *stx2a* gene at the same SBI, explains the enhanced pathogenicity associated with this clade and may represent an emerging, increased threat to public health. Supplementing routine analysis of short-read sequencing data with long-read sequencing analysis enables us to monitor the loss of, acquisition of and detection of multiple copies of *stx*-encoding bacteriophages, and improves our ability to predict emerging threats within the food chain, and provide accurate risk assessments during outbreak investigations.

## Methods

### Bacterial strains

There were 23 isolates of STEC O157:H7 PT21/28 in total, 20 linked to the outbreak in 2017, nine isolates were from six human cases, three from the RDM and eight from the cattle on the farm producing the milk^22^, and three isolates from two cases identified in 2019. All isolates belonged to sub-lineage Ic, had *stx2a* and fell within a unique five SNP single linkage cluster.

### DNA extraction, library preparation, Illumina sequencing and data processing

Genomic DNA was extracted from cultures of STEC O157:H7 using the QIAsymphony system (Qiagen, Hilden, Germany). The sequencing library was prepared using the Nextera XP kit (Illumina, San Diego, USA) for sequencing on the HiSeq 2500 instrument (Illumina, San Diego, USA), run with the fast protocol. FASTQ reads were processed using Trimmomatic v0.27^30^ as previously described^31^.

### DNA extraction, library preparation, Nanopore sequencing and data processing

High-molecular weight (HMW) genomic DNA was extracted and purified using the Revolugen Fire Monkey HMW DNA extraction kit (RevoluGen, UK), and DNA for each extract was quantified using a Qubit and the HS (high sensitivity) dsDNA assay kit (Thermofisher Scientific, Waltham, USA), as previously described^21,28^. Library preparation and sequencing was performed as previously described^28^ before sequencing on the MinION (Oxford Nanopore Technologies, Oxford, UK) for 48 h.

Data produced in a raw FAST5 format was base-called and de-multiplexed using Guppy v3.2.10 FAST model (Oxford Nanopore Technologies, Oxford, UK) into FASTQ format. De-multiplexing was performed using Deepbinner v0.2.0^32^, sequencing run metrics were generated using Nanoplot v1.8.1^33^, read trimming was performed using Porechop v0.2.4 (Wick RR, https://github.com/rrwick/Porechop)^34^ and finally, read filtering using Filtlong v0.2.0 (Wick RR, https://github.com/rrwick/Filtlong)^35^ as previously described^21,28^.

### De novo* assembly, correction, re-orientation and annotation.*

The filtered Nanopore FASTQ file with the 50× coverage of longest reads were assembled using Flye v2.8^36^ with the minimum overlap length (-m) set to 10,000 and the –meta component enabled. Correction (polishing) of the assemblies was performed in a three-step process. Firstly, using Nanopolish v0.11.3^17^, secondly, using Pilon v1.22^37^ and finally Racon v1.3.3^38^ as previously described^21,28,39^. As the chromosome from each assembly was circularised and closed, they were re-orientated to start at the *dnaA* gene (GenBank accession no. NC_000913) from *E. coli* K-12, using the –fixstart parameter in Circlator v1.5.5^40^. Prokka v1.13^41^ was used to annotate the final assemblies as previously described [21.28].

### Prophage detection, excision, comparison and generation of neighbour-joining trees

Prophage sequences were detected and extracted from each samples’ chromosome as described in Shaaban et al.^19^ and Yara et al.^21^. Prophage sequences were re-annotated using Prokka v1.13^41^. Mash v2.2^42^ was used to sketch (sketch length 1000, kmer length, 21) all extracted prophages in the samples sequenced in this study and all prophages found in the strain Sakai STEC O157:H7 reference genome (BA000007)^14^. This analysis was also performed on STEC O157:H7 PT21/28 genomes 9000 (CP018252) and 397,404 (CP043019). The pairwise Jaccard distance between the prophages was calculated and a neighbour joining tree computed for both *stx-*encoding prophages and non-*stx-*encoding prophages. Trees were visualised and annotated using FigTree v1.4.4 https://github.com/rambaut/figtree^43^. Prophages and chromosomes were also aligned using Easyfig v2.2.5^44^. Details for prophages from publicly available strains can be found in supplementary Table 1.

### In silico plasmid typing and characterisation

The plasmid replicon for each non-chromosomal contig within each sample’s finial assembly was compared to PlasmidFinder’s v2.1 Enterobacteriaceae reference database^45^. An alignment was generated using Easyfig v2.2.5^44^ relying on BLASTn v2.9^46^. BLASTn parameters used were minimum identity = 90% and minimum length hit = 100bp.

### Variant calling and masking, SNP typing and generation of phylogenetic trees

For reference-based variant calling both Illumina and Nanopore FASTQ reads were mapped to the Sakai STEC O157 reference genome (BA000007) using BWA v0.7.3^47^ and Minimap2 v2.17^48^ respectively with the use of Samtools v0.7.17^49^. VCFs were produced using GATK v2.6.5 UnifiedGenotyper^50^. Variants that had a high-quality SNP ([> 90% for Illumina] [> 80% for Nanopore] consensus, minimum depth 10×, MQ ≥ 30) in at least one isolate were extracted for further analysis. Any variants called at positions that were within the known prophages in Sakai were masked from further analyses. 5-methylcytosine positions were identified using Nanopolish V0.11.3^17^ and then masked from the Nanopore VCFs as described in Greig et al.^51^. The final number of positions masked was 1,189,993 bp, leaving a final reference of 4,308,457 bp.

The maximum likelihood phylogenetic tree was constructed by RAxML v8.1.17^52^ using an alignment generated from SnapperDB^12^ that recombination had been accounting for by Gubbins v2.00^53^. Visualisation of the phylogenetic tree was performed using FigTree v1.4.4^43^. To detect false positive/negative SNPs called by Illumina and Nanopore reads, discrepant variant positions between Illumina and Nanopore relative to the reference genome were extracted. The aforementioned variants and those that also had a lower-than-average mapping quality were then masked in the alignment.

### Detection and characterisation of chromosomal structural variation

Chromosome synteny was compared by aligning outbreak sample chromosomes using Easyfig v2.2.5^44^. Once samples in one chromosome were aligned, structural differences could be determined and further characterised using Artimis v18.1.0^54^.

To determine if there were multiple isoforms within each sample’s reads (FASTQ). The FASTQ for an outbreak sample in one isoform was aligned to a finalised assembly with a different isoform using Minimap2 v2.17^48^ and Samtools v0.7.17^49^. Using Tablet v1.17.08.17^55^, the alignments were visualised and the breakpoints at each isoform where identified. Once breakpoints were identified relative to each isoform, those positions were used with Samtools v0.7.17^49^ view to isolate reads that must align across both ends of each prophage breakpoint. Any reads that did align across a given set of breakpoints must share the same size as it exists in the FASTQ file and not clipped, to be considered.

### Data deposition

Illumina and Nanopore FASTQ files are available from National Center for Biotechnology Information (NCBI) BioProject PRJNA315192. The SRA (sequence read archive) accession numbers for both technologies are in supplementary Table 2. The outbreak sample finalised assemblies can also be found under BioProject PRJNA315192 and the GenBank accession numbers are located in both Table 2 and supplementary Table 2.

### Supplementary Information

Supplementary Table S1.

Supplementary Table S2.

## Data Availability

All FASTQ files and assemblies were submitted to the National Centre for Biotechnology Information (NCBI). All data can be found under BioProject: PRJNA315192 - https://www.ncbi.nlm.nih.gov/bioproject/?term=PRJNA315192. Strain-specific details can be found in Methods under data deposition.
