# Supplementary material for: Re-analysis of an outbreak of Shiga toxin-producing Escherichia coli O157:H7 associated with raw drinking milk using Nanopore sequencing
Source: Sci Rep. 2024 Mar 9;14:5821. doi: 10.1038/s41598-024-54662-0 (PMC10925052; doi:10.1038/s41598-024-54662-0)
Supplement: Supplementary file 1 — Supplementary Table S1. [file 41598_2024_54662_MOESM1_ESM.docx]

**Supplementary Tables:**

| **Strain ID** | **CC11 Sub-lineage** | **Phage Type** | ***Stx-*profile** | ***Stx1a prophage* SBI  and size (bp)** | ***Stx2a prophage* SBI  and size (bp)** | ***Stx2c prophage* SBI and size (bp)** | **Reference** | **Assembly Accession no.** |
| --- | --- | --- | --- | --- | --- | --- | --- | --- |
| **Outbreak genomes** | | | | | | | | |
| 413227 | Ic | PT21/28 | *Stx2a/2a* | - | *sbcB* (56,183) *argW* (66,541) | - | This study | CP088060-1 |
| 421196 | Ic | PT21/28 | *Stx2a/2a* | - | *sbcB* (56,429) *argW* (66,540) | - | This study | CP088058-9 |
| 423917 | Ic | PT21/28 | *Stx2a/2a* | - | *sbcB* (56,427) *argW* (66,536) | - | This study | CP088056-7 |
| 427603 | Ic | PT21/28 | *Stx2a/2a* | - | *sbcB* (56,435) *argW* (66,542) | - | This study | CP088071-2 |
| 429691 | Ic | PT21/28 | *Stx2a/2a* | - | *sbcB* (56,420) *argW* (66,537) | - | This study | CP088054-5 |
| 429692 | Ic | PT21/28 | *Stx2a/2a* | - | *sbcB* (56,426) *argW* (66,536) | - | This study | CP088052-3 |
| 429693 | Ic | PT21/28 | *Stx2a/2a* | - | *sbcB* (56,422) *argW* (66,540) | - | This study | CP088050-1 |
| 432297 | Ic | PT21/28 | *Stx2a/2a* | - | *sbcB* (56,150) *argW* (66,452) | - | This study | CP088048-9 |
| 432298 | Ic | PT21/28 | *Stx2a/2a* | - | *sbcB* (56,439) *argW* (66,539) | - | This study | CP088046-7 |
| 432299 | Ic | PT21/28 | *Stx2a/2a* | - | *sbcB* (56,427) *argW* (66,537]) | - | This study | CP088044-5 |
| 432300 | Ic | PT21/28 | *Stx2a/2a* | - | *sbcB* (56,430) *argW* (66,540) | - | This study | CP088042-3 |
| 432301 | Ic | PT21/28 | *Stx2a/2a* | - | *sbcB* (56,428) *argW* (66,539) | - | This study | CP088040-1 |
| 438729 | Ic | PT21/28 | *Stx2a/2a* | - | *sbcB* (56,441) *argW* (66,536) | - | This study | CP088069-70 |
| 432750 | Ic | PT21/28 | *Stx2a/2a* | - | *sbcB* (56,433) *argW* (66,538) | - | This study | CP088067-8 |
| 438602 | Ic | PT21/28 | *Stx2a/2a* | - | *sbcB* (56,379) *argW* (66,537) | - | This study | CP088038-9 |
| 435354 | Ic | PT21/28 | *Stx2a/2a* | - | *sbcB* (56,441) *argW* (66,539) | - | This study | CP088064-6 |
| 437021 | Ic | PT21/28 | *Stx2a/2a* | - | *sbcB* (56,429) *argW* (66,540) | - | This study | CP088062-3 |
| 437022 | Ic | PT21/28 | *Stx2a/2a* | - | *sbcB* (56,435) *argW* (66,543) | - | This study | CP088036-7 |
| 437023 | Ic | PT21/28 | *Stx2a/2a* | - | *sbcB* (56,427) *argW* (66,542) | - | This study | CP088034-5 |
| 437024 | Ic | PT21/28 | *Stx2a/2a* | - | *sbcB* (56,425) *argW* (66,538) | - | This study | CP088032-3 |
| 804533 | Ic | PT21/28 | *Stx2a/2a* | - | *sbcB* (56,433) *argW* (66,414) | - | This study | CP088030-1 |
| 811034 | Ic | PT21/28 | *Stx2a/2a* | - | *sbcB* (56,438) *argW* (67,194) | - | This study | CP088028-9 |
| 811035 | Ic | PT21/28 | *Stx2a/2a* | - | *sbcB* (56,405) *argW* (66,539) | - | This study | CP088026-7 |
| **Publicly available genomes** | | | | | | | | |
| Sakai | Ia | - | *Stx1a/2a* | *yehV* (47,650) | *wrbA* (62,142) | - | Michino *et al*. 1999 | BA000007 |
| E30228 | Ia | PT4 | *Stx1a/2a* | *yehV* (47,594) | *wrbA* (62,890) | - | Scotland *et al*. 1987 | VXJO00000000 |
| EDL933 | Ia | - | *Stx1a/2a* | *yehV* (47,596) | *wrbA* (61,066) | - | Riley *et al*. 1983 | CP008957 |
| 644 | IIc | PT8 | *Stx1a/1a/2c* | *yehV* (49,544) *argW* (64,569) | - | *sbcB* (58,210) | Cowley *et al*. 2016 | CP015831 |
| 180 | IIc | PT54 | *Stx1a/1a/2c* | *yehV* (49,544) *argW* (64,569) | - | *sbcB* (61,558) | Cowley *et al*. 2016 | CP015832 |
| TW14359 | I/IIa | - | *Stx2a/2c* | - | *argW* (71,540) | *sbcB* (60,476) | Uhlich *et al*. 2006 | NC_013008 |
| EC4115 | I/IIa | - | *Stx2a/2c* | - | *argW* (71,540) | *sbcB* (60,476) | Uhlich *et al*. 2006 | NC_011353 |
| 194195 | IIa | PT24 | *Stx2a/2c* | - | *argW* (74,662) | *sbcB* (60,586) | Greig *et al.* 2020 | CP044350 |
| E34500 | I/IIa | PT2 | *Stx2a/2c* | - | *argW* (62,149) | *sbcB* (57,463) | Taylor *et al*. 1986 | VXJN00000000 |
| 272 | I/IIa | PT2 | *Stx2a* | - | *argW* (65,675) | - | Jenkins *et al.* 2015 | CP018239 |
| E116508 | Ic | PT21/28 | *Stx2a/2c* | - | *argW* (71,870) | *sbcB* (59,105) | Yara *et al.* 2020 | VXJP00000000 |
| 397404 | Ic | PT21/28 | *Stx2a/2c* | - | *argW* (70,472) | *sbcB* (59,098) | Greig *et al.* 2021 | CP043019 |
| 350 | IIc | PT8 | *Stx1a/2c* | *yehV* (49,867) | - | *sbcB* (57,747) | Launders *et al*. 2016 | CP018243 |
| 824422 | IIa | PT34 | *Stx2a/2a/2c* | - | *rspA* (106,591) *yecE* (46,454) | *sbcB* (59,045) | Greig *et al*. 2020 | CP058231 |
| 818062 | IIa | PT34 | *Stx2a/2a/2c* | - | *rspA* (154,371) *yecE* (47,309) | *sbcB* (58,593) | Greig *et al*. 2020 | CP058233 |
| 155 | Ic | PT32 | *Stx2a* | - | *yecE* (50,015) | - | Shaaban *et al.* 2016 | CP018237 |
| 267849 | IIa | PT34 | *Stx2a/2c* | - | *yecE* (47,242) | *sbcB* (61,840) | Gobin *et al.* 2018 | VXJR00000000 |
| 315176 | IIb | PT8 | *Stx2a* | - | *sbcB* (61,851) | - | Byrne *et al*. 2018 | VXJQ00000000 |
| 9000 | Ic | PT21/28 | *Stx2a/2c* | - | *argW* (65,158) | *sbcB* (57,408) | Shaaban *et al.* 2016 | CP018252 |
| E45000 | I/IIb | PT49 | *Stx2a* | - | *sbcB* (44,014) | - | Yara *et al.* 2020 | VXJM00000000 |

**Table S1:** Summary of the publicly available genomes and sequenced genomes used within this study. Showing their Stain ID, CC11 sub-lineage, Phage Type, *stx* profile, the *stx*-encoding prophage associated with each sample including prophage size and SBI.
