# Supplementary material for: Re-analysis of an outbreak of Shiga toxin-producing Escherichia coli O157:H7 associated with raw drinking milk using Nanopore sequencing
Source: Sci Rep. 2024 Mar 9;14:5821. doi: 10.1038/s41598-024-54662-0 (PMC10925052; doi:10.1038/s41598-024-54662-0)
Supplement: Supplementary file 2 — Supplementary Table S2. [file 41598_2024_54662_MOESM2_ESM.docx]

| **Strain ID** | **NCBI Biosample accession** | **Illumina FASTQ SRA accession no.** | **Nanopore FASTQ SRA accession no.** | **Chromosome GenBank accession** | **Plasmid(s) GenBank accession(s)** |
| --- | --- | --- | --- | --- | --- |
| 413227 | SAMN08765101 | SRR6872965 | SRR16955622 | CP088060 | CP088061 |
| 421196 | SAMN08765099 | SRR6872964 | SRR16955621 | CP088058 | CP088059 |
| 423917 | SAMN08765102 | SRR6872963 | SRR16955610 | CP088056 | CP088057 |
| 427603 | SAMN08765095 | SRR6872960 | SRR16955606 | CP088071 | CP088072 |
| 429691 | SAMN08765089 | SRR6872954 | SRR16955605 | CP088054 | CP088055 |
| 429692 | SAMN08765088 | SRR6872956 | SRR16955604 | CP088052 | CP088053 |
| 429693 | SAMN08765098 | SRR6872961 | SRR16955603 | CP088050 | CP088051 |
| 432297 | SAMN08765092 | SRR6872958 | SRR16955602 | CP088048 | CP088049 |
| 432298 | SAMN08765096 | SRR6872953 | SRR16955601 | CP088046 | CP088047 |
| 432299 | SAMN08765087 | SRR6872959 | SRR16955600 | CP088044 | CP088045 |
| 432300 | SAMN08765091 | SRR6872950 | SRR16955620 | CP088042 | CP088043 |
| 432301 | SAMN08765097 | SRR6872957 | SRR16955619 | CP088040 | CP088041 |
| 438729 | SAMN08765100 | SRR6872962 | SRR16955611 | CP088069 | CP088070 |
| 432750 | SAMN08765093 | SRR6872951 | SRR16955618 | CP088067 | CP088068 |
| 438602 | SAMN09633761 | SRR7500962 | SRR16955612 | CP088038 | CP088039 |
| 435354 | SAMN08765094 | SRR6872952 | SRR16955617 | CP088064 | CP088065 + CP088066 |
| 437021 | SAMN09295082 | SRR7249752 | SRR16955616 | CP088062 | CP088063 |
| 437022 | SAMN09387732 | SRR7285585 | SRR16955615 | CP088036 | CP088037 |
| 437023 | SAMN08765090 | SRR6872955 | SRR16955614 | CP088034 | CP088035 |
| 437024 | SAMN09388585 | SRR7286572 | SRR16955613 | CP088032 | CP088033 |
| 804533 | SAMN12752398 | SRR10121279 | SRR16955609 | CP088030 | CP088031 |
| 811034 | SAMN12881735 | SRR10212174 | SRR16955608 | CP088028 | CP088029 |
| 811035 | SAMN12881788 | SRR10212179 | SRR16955607 | CP088026 | CP088027 |

**Table S2:**  Table detailing the Illumina and Nanopore NCBI SRA accessions and NCBI GenBank accession(s) for finalised assemblies.
